# Selecting Subpopulations of High-Quality Protein Conformers among Conformational Mixtures of Recombinant Bovine MMP-9 Solubilized from Inclusion Bodies

**DOI:** 10.3390/ijms22063020

**Published:** 2021-03-16

**Authors:** Jose Vicente Carratalá, Laia Gifre-Renom, Ramon Roca-Pinilla, Antonio Villaverde, Anna Arís, Elena Garcia-Fruitós, Julieta María Sánchez, Neus Ferrer-Miralles

**Affiliations:** 1 Institute for Biotechnology and Biomedicine, Autonomous University of Barcelona, Bellaterra, 08193 Barcelona, Spain; josevicente.carratala@uab.cat (J.V.C.); Antoni.villaverde@uab.cat (A.V.); 2 Department of Genetics and Microbiology, Autonomous University of Barcelona, Bellaterra, 08193 Barcelona, Spain; 3 Bioengineering, Biomaterials and Nanomedicine Networking Biomedical Research Centre (CIBER-BBN), Autonomous University of Barcelona, Bellaterra, 08193 Barcelona, Spain; 4 Department of Ruminant Production, Institute of Agrifood Research and Technology (IRTA), Caldes de Montbui, 08140 Barcelona, Spain; laiagifrerenom@gmail.com (L.G.-R.); ramon.rocap@gmail.com (R.R.-P.); anna.aris@irta.cat (A.A.); elena.garcia@irta.cat (E.G.-F.)

**Keywords:** inclusion bodies, affinity chromatography, dynamic light scattering, the center of spectral mass, circular dichroism, protein conformers

## Abstract

A detailed workflow to analyze the physicochemical characteristics of mammalian matrix metalloproteinase (MMP-9) protein species obtained from protein aggregates (inclusion bodies—IBs) was followed. MMP-9 was recombinantly produced in the prokaryotic microbial cell factories *Clearcoli* (an engineered form of *Escherichia coli*) and *Lactococcus lactis,* mainly forming part of IBs and partially recovered under non-denaturing conditions. After the purification by affinity chromatography of solubilized MMP-9, four protein peaks were obtained. However, so far, the different conformational protein species forming part of IBs have not been isolated and characterized. Therefore, with the aim to link the physicochemical characteristics of the isolated peaks with their biological activity, we set up a methodological approach that included dynamic light scattering (DLS), circular dichroism (CD), and spectrofluorometric analysis confirming the separation of subpopulations of conformers with specific characteristics. In protein purification procedures, the detailed analysis of the individual physicochemical properties and the biological activity of protein peaks separated by chromatographic techniques is a reliable source of information to select the best-fitted protein populations.

## 1. Introduction

Recombinant proteins are obtained from a wide collection of microbial expression systems [1,2]. However, in some instances, the recombinant protein ends up accumulated in the insoluble cell fraction [3,4]. These protein aggregates or nanoclusters (NCs), known as inclusion bodies (IBs) in prokaryotic expression systems, are complex structures stabilized by protein–protein cross β-sheet interactions forming a protease-resistant scaffold, which coexist with internalized native and native-like conformers of the protein of interest [5,6,7]. The complete denaturation of the aggregates and the subsequent refolding of the released protein species has been a widely used protocol for the isolation of soluble proteins using IBs as the protein source [8,9]. However, this approach often results in variable efficiency in the recovery of correctly folded proteins and, the biological activity may be highly compromised [10]. In the last decades, the detection of biological activity in these protein NCs fueled the development of alternative soluble protein purification procedures from IBs using non-denaturing conditions [11,12]. Taking into account the porous nature of these aggregates, the native and native-like species of the protein of interest may be separated from the scaffold structure by controlled release of soluble conformers through incubation with buffers containing mild detergents at low concentrations [13]. The resulting protein solution, enriched with the protein of interest in a soluble format, can be then easily separated from the aggregated remnants of IBs by centrifugation [11,13,14]. In addition, it might contain a wide range of folding intermediates with a dissimilar specific activity. In order to demonstrate the presence of this spectrum of protein species in the protein aggregates and with the aim to select protein subpopulations with the best conformational quality, we selected matrix metalloproteinase 9 (MMP-9) as a paradigm of difficult-to-produce eukaryotic protein in prokaryotic expression systems [15]. Matrix metalloproteinases (MMPs) constitute a family of zinc-dependent enzymes involved in the degradation and remodeling of the extracellular matrix. In addition, MMP-9 seems to play important roles in tissue reorganization in physiological processes, including embryogenesis, neovascularization and in the course of the restructuring of synaptic connections [16,17].

In this study, two endotoxin-free prokaryotic expression systems, *Clearcoli*^®^ BL21(DE3) and *Lactococcus lactis* were transformed with an expression vector containing a His-tagged version of the bovine MMP-9 gene to compare for the ability of the corresponding protein folding machinery to cope with the product of the overexpressed gene. MMP-9 was produced mostly in the form of IBs in both expression systems, and mild detergent treatment was performed to release entrapped protein, as reported [18]. The results showed that in both cases, four different peaks were obtained after affinity chromatography analysis indicating the presence of several subpopulations of conformers with variable ability to interact and coordinate to the Ni^2+^ of the resin. The specific activity of the resulting protein peaks appeared to be related to higher helical content in the structure. In addition, the results linked the presence of more compact conformations to higher thermal stability. We expect that this type of analysis will be useful for understanding the conformational complexity of IB proteins and selecting the best-fitted population of native-like containing conformers from a complex mixture of protein species released from IBs under non-denaturing conditions.

## 2. Results and Discussion

### 2.1. Protein Production of Soluble MMP-9 in IBs

Mammalian MMP-9 is an aggregation-prone protein when recombinantly produced in prokaryotic expression systems, such as *E. coli and L. lactis*, being necessary to purify the soluble version from IBs [19]. Thus, the soluble form of prone-to-aggregate proteins, such as MMP-9, can only be obtained from bacterial IBs by using denaturing or non-denaturing procedures [11,13,20,21,22,23]. Recovering protein species in an active state by refolding protocols from denatured proteins is time-consuming and results in variable performance efficiency. For these reasons and based on the discovery of bioactive protein conformations as an important IB component, the application of non-denaturing solubilization protocols has become a promising alternative [12,13,21]. However, the heterogeneous nature of the protein forms released from IBs has not been studied. Herein, we have analyzed the different active conformers derived from the protein pool obtained after solubilizing MMP-9 protein from IBs of two generally recognized as safe (GRAS) microorganisms (*Clearcoli* and *L. lactis*). In both cases, the protein was primarily detected in the insoluble cell fraction as expected (data not shown). In the case of *Clearcoli*, the expression of the recombinant gene had a clear negative effect on the overall fitness of the cultures since the final OD_550_ of the cultures remained at the same level or slightly higher than the initial pre-induction values (Appendix A, Table S1).

### 2.2. Solubilization of Recombinant MMP-9 from IBs

**Figure 1:**
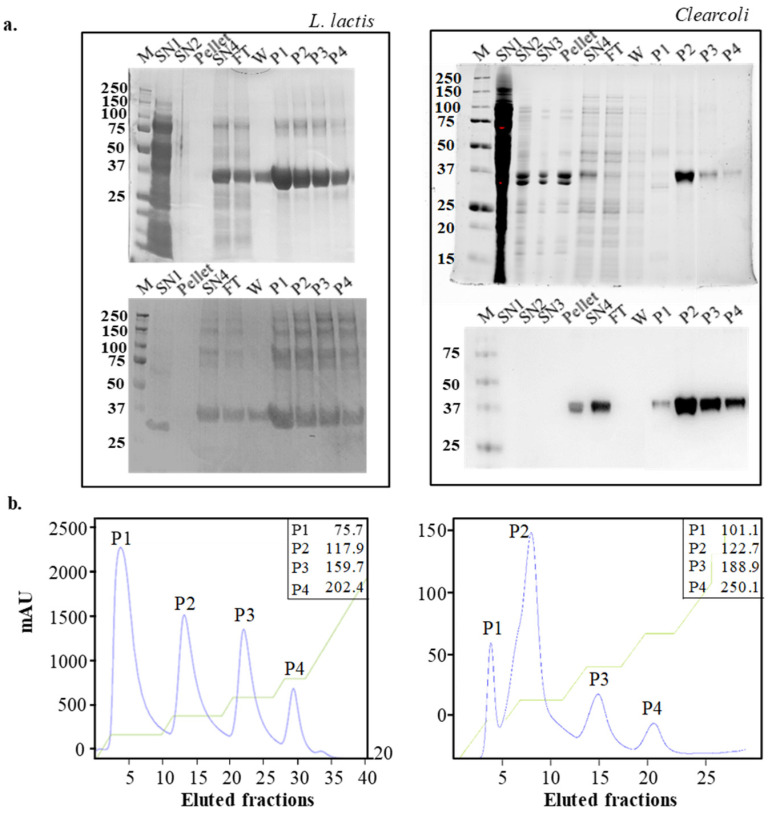
Detection of protein bands from inclusion bodies (IBs) produced in *L. lactis* (left) and *Clearcoli* (right) obtained during solubilization procedure. For each expression system, SDS–PAGE (above) and Western blot analyses (below) are shown (**a**). Immobilized metal affinity chromatography (IMAC) chromatograms for purifications of solubilized MMP-9 samples produced by *L. lactis* (left) and *Clearcoli* (right). Blue lines depict the absorbance signal (mAU) along the elution process and green lines the elution buffer (EB) gradient progress. The corresponding concentration of imidazole (mmol/L) is indicated for each eluted peak in the inset (**b**). SN1: soluble cell fraction of the cell lysate; SN2: soluble protein content after the first wash of the insoluble cell fraction; SN3: soluble protein content after second wash; pellet: pellet after solubilization of IBs with N-lauroylsarcosine; SN4: proteins solubilized from IBs after N-lauroylsarcosine treatment; FT: Flow-through; W: wash; P1-P4: protein peaks. M: molecular weight marker in kDa.

Solubilization of MMP-9 from IBs of *L. lactis* and *Clearcoli* is shown in Figure 1a. Despite the release of the recombinant protein during the processing of the IB samples (see lanes Pellet (the content of IBs after resolubilization), SN1 (soluble cell fraction), SN2 (first washing of IBs), and SN3 (second washing of IBs)), a significant amount of the protein was detected in the soluble fraction in SN4 (solubilized proteins from IBs) after incubation with solubilization buffer (containing 0.2% N-lauroylsarcosine) of both *L. lactis* and *Clearcoli* IBs. During the solubilization step, the anionic detergent interacts with MMP-9 through its hydrophobic tail. Detergents might form micelles when achieving the critical micelle concentration (CMC), inducing the denaturation of the protein. However, the CMC of N-lauroylsarcosine is 14.6 mmol/L (ref Bagheri 2019), which was not reached in the tested conditions at 6.8 mmol/L (0.2%). Some protein bands of an apparent molecular weight similar to MMP-9 were detected in the SN2, SN3 and Pellet samples in *Clearcoli*. However, in Western blot analysis, those protein bands were not identified as MMP-9. Although the efficiency of protein solubilization from IBs was variable [13] in the expression systems evaluated here, a substantial proportion of MMP-9 protein in IBs was released, allowing further purification.

### 2.3. Purification of Recombinant MMP-9 by Affinity Chromatography

**Figure 2:**
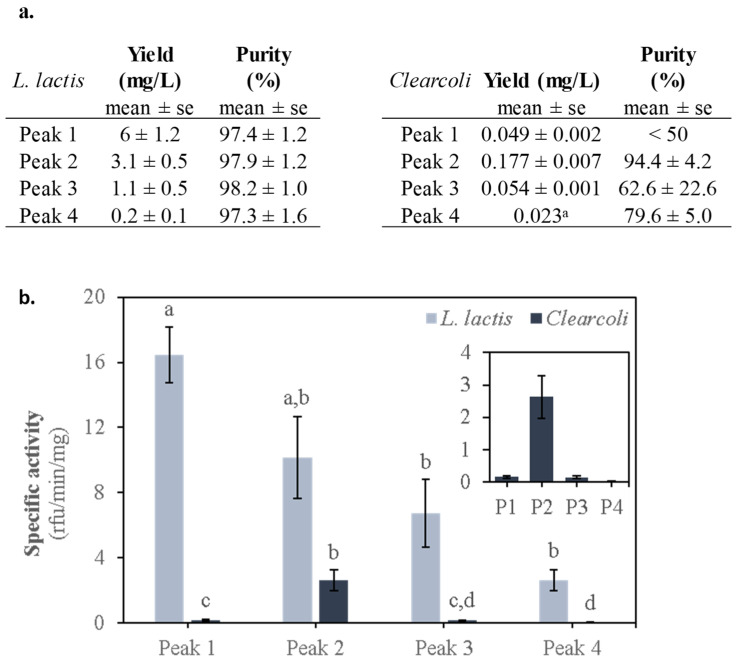
Table summarizing the yields for each peak, as mg of protein per peak per culture L (mg/L), and their purity in % for each expression system. ^a^ Peak 4 of *Clearcoli* was purified in enough amount to be quantified only once out of the 3 separate production experiments performed. Therefore, no SEM is shown (**a**). Specific activity for the MMP-9 in each peak solubilized from IBs produced by *L. lactis* and *Clearcoli*. Relative fluorescence units (rfu) refer to the fluorescence emitted by dye-quenched gelatin along its degradation kinetics due to MMP-9 activity. Specific activity is expressed as rfu per minute per MMP-9 mg (rfu/min/mg). Means and standard error of the mean (SEM) are depicted for each MMP-9 peak (*n* = 4). Different letters (a to d) depict differences between protein peaks (*p* = 0.002) (**b**).

Affinity chromatography of the solubilized MMP-9 from *L. lactis* and *Clearcoli* generated four protein peaks containing MMP-9 (Figure 1b). The identity of MMP-9 was observed in each of the protein peaks (Figure 1a, lower panels). It has been described that recombinant MMP-9 forms mixtures of monomers with higher oligomeric species [24], and positive protein bands of high molecular weight were observed at least in the protein samples purified from *L. lactis*, indicative of the presence of oligomers. The low total amount of purified protein from *Clearcoli* was not enough to reveal the presence of oligomers under the tested experimental conditions. The presence of different protein peaks in the pool of solubilized protein indicates the coexistence of several protein conformers with dissimilar affinities for the Ni^2+^-loaded resin. The variable affinity of the protein forms for the columns may be due to local conformational changes of the His-tag or to the presence of protein conformers with dissimilar occupancy of the seven Zn^2+^-binding sites in the protein (UniProt P52176; Figure S2). The empty binding sites may interact with the coordinated Ni^2+^ displayed on the resin [25]. On the other hand, we have detected more than one elution peak in IMAC chromatography by using similar IB solubilization protocols in other families of proteins apart from metalloproteinases, suggesting that the presence of active folding intermediates in the solubilization mixture from IBs is not exclusive for metal-containing proteins (Figure S3). However, we cannot rule out the possibility that in the case of MMP-9, the distinct level of occupancy of the metal-binding sites may affect the distribution of conformational populations. In addition, we can consider the possibility that each protein peak was stabilized by a unique interaction with the detergent. In any case, irrespectively of the final yield of protein recovery in each of the peaks (compare the peak height for the two expression systems and the final yield in Figure 1b and Figure 2a, respectively), a multi-peak elution profile was obtained for both expression systems (Figure 1b). However, the purity between the equally numbered protein peaks was not homogeneous. In fact, peak 1 obtained from *Clearcoli* included a great proportion of contaminant proteins and was discarded for further analysis (Figure 1a). Another case to mention was peak 3 of *Clearcoli* (62. 5% purity), which was still included in protein characterization experiments as the contaminant protein bands were also detected in protein peak 2 of *Clearcoli,* considering then a similar interference between protein samples. In addition, equivalent peaks from each expression system eluted at different imidazole concentrations (Figure 1b insets), indicating that they may correspond to distinguishable protein conformational populations between both expression systems [26]. Several attempts were made to purify the low quantity of recombinant protein accumulated in the soluble cell fraction to obtain a quality control reference. However, this protein version was difficult to purify and had a great tendency to aggregate under the tested experimental conditions.

### 2.4. Activity of the MMP-9 Protein Peaks of L. Lactis and Clearcoli Obtained from IBs

The highest activity of *L. lactis* protein peaks corresponded to peak 1, although no significant differences were detected between peaks 1 and 2. Moreover, significant differences were obtained between peaks 1 and peaks 3 and 4 (*p* = 0.0002) (Figure 2b). On the other hand, *Clearcoli* protein peak 2 was the only one showing activity in this expression system (*p* = 0.0002). In any case, the activity of each of the *L. lactis* protein peaks was significantly higher than that of any of the protein peaks obtained from *Clearcoli.* This observation supports the potential of this expression system as a promising alternative to *E. coli* for the production of recombinant proteins [27]. These results clearly stressed that the selection of this prokaryotic expression system has a clear impact on the final quality of the produced recombinant protein.

### 2.5. Physicochemical MMP-9 Properties

In the biopharmaceutical industry, the biophysical characterization of therapeutic proteins follows a rigorous and standardized process [28]. In addition, some specific physicochemical methods are being established in protein structure studies [29]. However, in many research laboratories, access to specialized equipment and trained personnel is not common. In this case, the detection of more than one positive protein peak during the purification process is not evaluated under the parameters of conformational quality. In that sense, we selected some available methodological approaches to analyze the putative correlation between protein conformational quality and biological activity in the different protein peaks obtained during the purification process of MMP-9.

**Figure 3:**
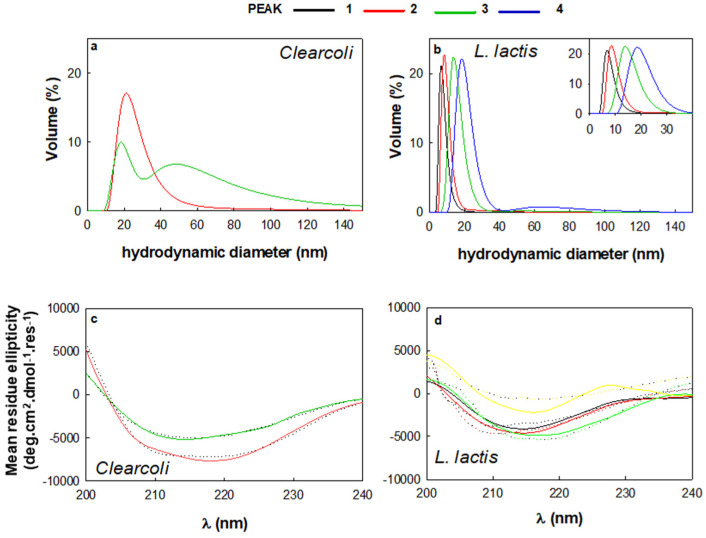
Volume weighted distribution determined by dynamic light scattering (DLS) of MMP-9 peaks from, *Clearcoli* (**a**) and *L. lactis* (**b**). Far UV-circular dichroism (CD) spectra of MMP-9 peaks from *Clearcoli* (**c**) and *L. lactis* (**d**). Experimental spectra (dotted lines) and fitted spectra (solid lines). (See Materials and Methods Section 3.8).

Interestingly, we detected a correlation between the affinity of the protein subpopulations towards the Ni^2+^ (i.e., higher peak number in Figure 1b) and the size of the protein species revealed by DLS (Figure 3a,b and Table 1), that is irrespective of the bacterial strain.

In *L. lactis*, the protein size moves from 6–8 nm to approximately 20 nm or more, suggesting an oligomerization event. In fact, according to the Wilkins equation [30], the hydrodynamic diameter of recombinant protein MMP-9 in native corresponds to 5.2 nm, which is close to the size detected for peak 1 in *L. lactis* (Table 1). However, the enzyme functionality was also detected in samples of slightly higher hydrodynamic diameter (as peak 2 from *L. lactis*). On the other hand, the hydrodynamic diameter calculated with the same equation for the unfolded protein is 12.4 nm. Surprisingly, protein samples with similar hydrodynamic diameters displayed enzymatic activity (protein peaks 3 and 4 of *L. lactis* and peak 2 of *Clearcoli*). Therefore, the higher dimensions of protein peaks could be explained by the presence of higher proportions of disordered structures (Table 2) rather than a full unfolding process, at least in the case of protein peaks 3 and 4 of *L. lactis*. In addition, as the size of the protein increases, the presence of alpha structure seems to fade away (Figure 3d, Table 2), suggesting a link between oligomerization and the secondary structure of the protein. In this context, MMP-9 from peaks 1 and 2 for *L. lactis* and peak 2 for *Clearcoli* exhibited a higher percentage of α-helix structure (a particularly noticeable minimum at around 208 nm (*L. lactis*) or at 210 and 222 nm (*Clearcoli*) that tends to disappear in peaks 3 and 4 (Table 2). Moreover, MMP-9 from peak 3 (*Clearcoli*), which contains more than one peak in the DLS analysis, revealed a β-sheet protein spectrum, as an incipient minimum around 216 nm was observed (Figure 3a and Table 2). Such protein conformational change concomitant with the oligomerization process has been previously described during the controlled protein assembly as regular size protein nanoparticles [31]. However, it cannot be ruled out that the increase in the size of the proteins might be due to the presence of disordered structures (Table 2).

Another important parameter for the enzymatic activity of MMP-9 is the presence of metal ions. Therefore, in order to assess the Zn^2+^ occupancy in the protein conformers present in the protein peaks, inductively coupled plasma mass spectrometry (ICP-MS) was performed (Appendix A and Figure S4) as previously described [32]. Overall, the Zn^2+^ occupancy in all protein samples was below the expected molar ratio for this recombinant protein with 7 putative binding sites (Figure S2A). Surprisingly, the amount of Zn^2+^ was much lower for the protein samples obtained in *L. lactis* (Figure S4A), which corresponds to the expression system where the maximum specific activity of MMP-9 was achieved (*L. lactis* protein peak 1 and 2, see Figure 2b). In addition, in *Clearcoli*, protein peak 2, which displayed the highest specific activity (Figure 2b), contained the lowest amount of Zn^2+^ (Figure S4A). Even though the presence of metal ions is relevant for the biological activity of enzymes, in controlled experimental conditions, it has been described a negative impact on the specific activity relative to the metal ion concentration [33]. In the results presented here, most of the protein samples presented less than 7 Zn^2+^ ions per protein molecule, except for protein peak 3 from *Clearcoli* (Figure S4A). Moreover, data obtained for Ni^2+^ showed a similar trend, detecting much more signal in *Clearcoli* than in *L. lactis,* and with a concomitant increase in the amount of this cation while increasing the number of the protein peak (Figure S4B). However, when the total amount of metal ions was calculated, a clear proportional relationship between cation levels and protein peak number was observed (Figure S4C,D). One possible explanation of the detection of enzymatic activity on protein peaks with low content of metal cations would be the direct relationship between the presence of metal ions and the oligomerization state of protein samples [34,35]. In fact, DLS results indicated an increase in size for protein peaks with lower enzymatic activity (Figure 3).

**Figure 4:**
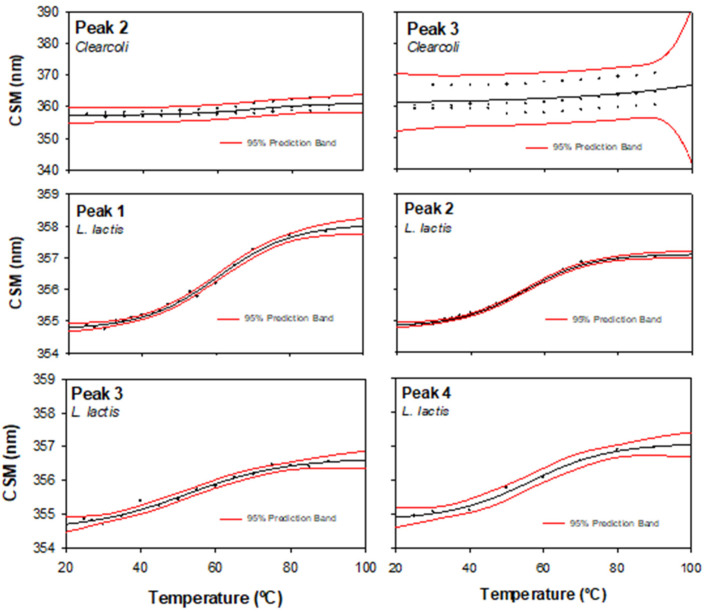
Center of spectral mass of the tryptophan fluorescence spectrum (CSM) versus temperature of each MMP-9 peak (indicated in the plot). The solid line indicated the nonlinear regression to a sigmoidal model; red lines indicated 95% of the prediction interval.

Another structural parameter, namely CSM, was obtained from the intrinsic fluorescence (IF) spectra. This value is related to the tertiary structure of the protein, and its increase indicates the hydration of the whole structure that, in many cases, accompanies protein unfolding. Figure 4 and Table 1 show the CSM values from each protein peak and from both bacterial hosts. Neither the supramolecular structure (DLS) nor the secondary structure (CD) allowed us to appreciate the differences between the peak-corresponding proteins produced in each expression system. However, at 25 °C, CSM values from *L. lactis* were around 354 nm with modest variability, while the ones from *Clearcoli* exhibit higher CSM values (Table 1 and Figure 4), which was probably related to the loss of the tertiary structure. Moreover, it is important to highlight the high variability in the CSM values observed when MMP-9 was obtained from *Clearcoli*.

In Figure 4, we show the CSM thermal profile of each MMP-9 peak (raw data exemplified in Supplementary Material Figure S4) and the statistical estimation of the Tm values recorded in Table 1. As we discuss below, the highest structure-function quality and, at the same time, lower variability on the estimation were observed in samples from peak 2. In the case of *Clearcoli*, the sample from peak 2 was the only one that showed reliable data.

In biophysical terms, the native state of proteins is described as that of minimum energy with a limited number of conformational structures (or low conformational entropy) [36]. However, in many cases, the unfolded to native form transition occurs by a multistep folding process with the generation of conformation intermediates [37,38]. In this sense, the structure variability detected in different positive MMP-9 protein peaks after IB solubilization is in accordance with the presence of folding intermediates entrapped in the IBs irrespectively of the expression system. In addition, after the detailed physicochemical analysis of the individual protein peaks, we can conclude that the second protein peak contained the protein in its best conformational state and presents the lowest variability regardless of the bacterial producing system. According to this analysis, the MMP-9 protein species from peak 2 produced in *L. lactis* contained the most structured and functional protein conformers.

## 3. Materials and Methods

### 3.1. Bacteria Strains and Plasmids

The *Lactococcus lactis* subsp. *cremoris* NZ9000 mutant (*clpP^−^*, *htrA^−^*, Em^R^) strain, provided by INRA (France; patent n. EP1141337B1) was transformed with a pNZ8148 plasmid with chloramphenicol resistance gene (Cm^R^) (MoBiTech GmbH, Goettingen, Germany) previously cloned with the DNA insert encoding for a bovine MMP-9 fragment (Phe107-Pro449, NCBI, NM_174744.2) [15].

The same DNA fragment encoding for the bovine Phe107-Pro449 MMP-9 was cloned (*Nco*I/*Hind*III restriction sites) into a pETDuet plasmid (Novagen, Madison, WI, USA) bearing the ampicillin resistance gene (Am^R^) and transformed in *Clearcoli*^®^ BL21(DE3) (Lucigen, Middleton, WI, USA) by electroporation (Appendix A).

The DNA fragment in both expression vectors was C-terminally fused to a 6His-tag and codon-optimized for *L. lactis* (GeneArt, Thermo Fisher, Waltham, MA, USA; Figure S1) [15].

### 3.2. Bacteria Strains and Plasmids

Batch cultures of *L. lactis* were grown at 30 °C in static cultures with M17 broth containing 0.5% glucose, 5 µg/mL chloramphenicol (Cm) and 2.5 µg/mL erythromycin (Em). Inductions of re-inoculated cultures were done with 12.5 ng/mL nisin at 0.4–0.6 OD_600_ for 3 h to get the recombinant protein expression.

Batch cultures of *Clearcoli* were grown at 37 °C in a shaker at 250 rpm with lysogeny broth (LB; 10 g/L tryptone, 5 g/L yeast extract, 10 g/L NaCl) and 100 µg/mL ampicillin. Inductions of re-inoculated cultures were done at 1 mmol/L IPTG when cell suspensions reached 0.6–0.8 OD_550_. The cultures were then incubated at 30 °C and 250 rpm for 3 h (for protein production). Bacteria were harvested by centrifugation at 6000× *g* for 30 min at 4 °C.

### 3.3. Protein Purification

Soluble MMP-9 was obtained by protein solubilization from IBs as described [11]. For each solubilization process, five samples were generated: SN1 (i.e., supernatant 1, soluble cell fraction of the lysate), SN2 and SN3 from washes of the insoluble cell fraction after cell lysis, SN4 and the cell pellet obtained after solubilization with mild detergent incubation. The MMP-9 protein contained in SN4 was purified by affinity chromatography in an ÄKTA Pure fast protein liquid chromatography (FPLC) system (GE Healthcare, Chicago IL, USA) (Appendix A).

### 3.4. Protein Detection, Yield and Purity

The different protein fractions collected after the purification process were analyzed by SDS–PAGE and Western blot analyses. Briefly, a small aliquot of each fraction was independently mixed (1:1) with Lemmli buffer. Then, the different samples were boiled at 90 °C for 10 min and subsequently charged in a polyacrylamide gel. Samples containing aggregated protein (pellets) were boiled for 40 min. The electrophoresis was run in a buffer containing 0.1% of sodium dodecyl sulfate (SDS).

Positive protein bands were detected by Western blot. The conditions used were anti-his-tag monoclonal primary antibody (Santa Cruz Biotechnologies, Inc., Santa Cruz Biotechnologies, Inc., Dallas, TX, USA; scv-57598) used at 1:1000 dilution, and 6xhis monoclonal antibody (Takara Bio Inc., Kusatsu, Japan; 631212) used at 1:6000 dilution for *L. lactis* and *Clearcoli,* respectively, and goat anti-mouse secondary antibody at 1:5000 dilution (Bio-Rad Laboratories Inc., Hercules, CA, USA; 170–6516). The images were acquired with the ChemiDoc™ touch imaging system (Bio-Rad Laboratories Inc., Hercules, CA, USA).

Soluble MMP-9 in each peak was quantified by NanoDrop (Thermo Fisher Scientific, Waltham, MA, USA) using the MMP-9 parameters (ε: 70,080 mol/L^−1^ cm^−1^; ProtParam-ExPASy) and yield for each peak was obtained. The purity of MMP-9 peaks was analyzed by Coomassie blue or TGX (Bio-Rad Laboratories Inc., Hercules, CA, USA) staining using ImageLab software version 6.1.0 (Bio-Rad Laboratories Inc., Hercules, CA, USA). Briefly, measurements of the volume of the protein bands in each lane were considered as 100% of the protein content in the sample, and the volume of the protein band corresponding to MMP-9 was used to calculate the percentage it represents in the total protein bands in the lane.

### 3.5. MMP-9 Activity Determination by DQgelatinTM Degradation Kinetics

MMP-9 activity for each eluted peak from both *L. lactis* and *Clearcoli*^®^ BL21(DE3) productions was quantified by dye-quenched gelatin (DQgelatin™, Thermo Fisher Scientific, Waltham, MA, USA) degradation kinetics (Appendix A). Specific activity for MMP-9 peaks was extracted for each sample by obtaining the initial velocity from the kinetics data (relative fluorescence units per minute, rfu/min) and correcting it by the MMP-9 mg in the wells (rfu/min/mg).

### 3.6. Dynamic Light Scattering (DLS)s

The volume size distribution of MMP-9 from each chromatographic peak was determined at 0.15 mg/mL in 20 mmol/L Tris-HCl pH 8 and 5% glycerol by DLS at 633 nm (Zetasizer Nano ZS, Malvern Instruments Limited, Malvern, UK). Samples were maintained at 25 °C. According to the Stokes–Einstein equation, the DLS algorithm calculates the hydrodynamic radius or hydrodynamic diameter from the diffusion coefficient of the particles [39].

### 3.7. Determination of Intrinsic Fluorescence

Fluorescence spectra were recorded in a Cary Eclipse spectrofluorometer (Agilent Technologies, Mulgrave, Australia). A quartz cell with 10 mm path length and a thermostated holder was used. The excitation and emission slits were set at 5 nm. Excitation wavelength (λ_ex_) was set at 295 nm. Emission spectra (λ_em_) were acquired within a range from 310 to 450 nm. The protein concentration was around 0.2 mg/mL in 20 mmol/L Tris-HCl pH 8 and 5% glycerol. Spectrum from each peak and of each bacterial strain was performed in triplicate. In order to evaluate the conformational difference between the proteins of each peak, we decided to calculate the center of spectral mass (CSM) for comparisons. CSM is a weighted average of the fluorescence spectrum peak. In addition, it is related to solvent exposure of the Trp. The maximum red-shift in the CSM of the tryptophan is compatible with a large solvent [20,40,41] and consequently a highly unfolded conformation.

The CSM was calculated for each of the fluorescence emission spectrum [42] according to Equation (1), where *I_i_* is the fluorescence intensity measure at the wavelength *λ_i_*.

(1) $$\lambda =\frac{\sum^{\;}\lambda_{i}I_{i}}{\sum^{\;}I_{i}\;}$$

We also performed thermal unfolding analyses by measuring Trp fluorescence as a function of the temperature. For this approach, the heating rate was set at 1 °C/min.

### 3.8. Circular Dichroism (CD)

Measurements were made with a Jasco J-715 spectropolarimeter (JASCO, Oklahoma City, OK, USA) with a thermostated device by a Peltier system spectropolarimeter using a 1 mm path length quartz cell. Each spectrum was an average of six scans. The protein concentration was around 0.1–0.2 mg/mL in 20 mmol/L Tris-HCl pH 8 and 5% glycerol. Scan speed was set at 50 nm/min with a 1 s response time, and measurements were carried out in the 200–240 nm region. Each final spectrum was obtained from two or three replicas. The ellipticity values were transformed in “mean residue ellipticity” as previously described [31]. The relative secondary structure contents of the protein from each peak was obtained by deconvoluting its far-UV CD spectrum by using the CONTIN-LL algorithm [43,44] run on the DichroWeb server [45].

### 3.9. Statistical Analysis

Data for the determination of MMP-9 activity by DQgelatin™ degradation kinetics were analyzed using a mixed-effects model, using SAS 9.4 (SAS Institute Inc., Cary, NC, USA). Replicates (*n* = 12 for peaks 2 and 3 obtained in *Clearcoli*; n = 9 for all peaks in *L. lactis*, and for peak 1 in *Clearcoli*; *n* = 4 for peak 4 in *Clearcoli*) were included as a random effect; strain, peak and their interaction were included as fixed effects. Differences between multiple means were further established using Tukey’s test. Data were previously transformed to a natural logarithm to achieve a normal distribution when needed. Results are expressed as means and standard error of nontransformed data.

The values of melting temperature (Tm) were determined by fitting the experimental data from the CSM versus temperature plot to a sigmoidal equation of four parameters by a computer-aided nonlinear regression analysis by the least-squares method.

In order to evaluate the variability of CSM values within the thermal profile, we showed the 95% prediction interval. This region illustrates the standard deviation of experimental data with respect to the estimated value.

## 4. Conclusions

Many recombinant proteins used for biopharmaceutical and industrial purposes are obtained from IBs. Despite the development of different protocols for the recovery of functional proteins from these aggregates, there is an unmet need for analytical methods to evaluate the conformational and functional status of the proteins released from IBs. In this study, the physicochemical analysis of MMP-9 protein peaks rescued from IBs of two endotoxin-free prokaryotic expression systems revealed the presence of different pools of protein conformers with specific structural characteristics. The oligomeric status of these protein forms, together with the content in alpha helices and the corresponding thermal stability, had an impact on the specific activity of the protein pools. This type of analysis can provide comprehensive views of the conformational heterogeneous nature of the folding intermediates released from IBs as well as allow for the rational selection of the best-fitted populations of protein forms.

## Figures and Tables

**Table 1:** DLS, CSM and unfolding temperature of isolated elution protein peaks obtained from *L. lactis* and *Clearcoli* IBs after solubilization. Values represent mean and SEM. Not determined (n.d.). Polydispersity index (pdi).

	Peak 1	Peak 2	Peak 3	Peak 4
**Hydrodynamic diameter (nm)**				
*Clearcoli*	n.d.	21 ± 9	18.2 ^1^ ± 5.3	n.d.
		(pdi = 0.6)	(pdi = 0.47)	
*L. lactis*	6.5 ± 2.1	8.04 ± 3.2	15.7 ± 4.1	18.2 ± 4.1
	(pdi = 0.7)	(pdi = 0.5)	(pdi = 0.7)	(pdi = 0.7)
**Center of spectral mass (CSM, nm)**				
*Clearcoli*	n.d.	357 ± 0.5	361 ± 9	n.d.
*L. lactis*	354.82 ± 0.1	354.89 ± 0.07	354.75 ± 0.2	354.92 ± 0.3
**Unfolding temperature (Tm, °C)**				
*Clearcoli*	n.d.	69.54 ± 8.2	n.d.	n.d.
*L. lactis*	60.6 ± 1.1	54.8 ± 0.7	52.4 ± 3.3	56.4 ± 2.5

^1^ This value corresponds to the smaller peak shown in Figure 3. The second peak (around 50 nm) displayed a very broad size distribution.

**Table 2:** Secondary structure contents of the MMP-9 protein obtained by deconvoluting far-UV CD spectra.

	Peak 1	Peak 2	Peak 3	Peak 4
*Clearcoli*				
**Alpha helix**	n.d.	0.242	0.132	n.d.
**Beta sheet**	n.d.	0.273	0.336	n.d.
**Turns**	n.d.	0.217	0.213	n.d.
**Disordered**	n.d.	0.266	0.319	n.d.
**NRMSD**	n.d.	0.095	0.057	n.d.
*L. lactis*				
**Alpha helix**	0.091	0.125	0.06	0.039
**Beta sheet**	0.389	0.377	0.349	0.409
**Turns**	0.197	0.198	0.197	0.196
**Disordered**	0.323	0.3	0.394	0.356
**NRMSD**	0.491	0.411	0.176	0.498

NRMSD: normalized root means square deviation.

## Data Availability

The data presented in this study are available on request from the corresponding author. The data are not yet publicly available because the deposit and review process has not yet been completed.

## Supplementary Materials

The following are available online at https://www.mdpi.com/1422-0067/22/6/3020/s1, Figure S1: *L. lactis* codon-optimized DNA encoding sequence of the cloned Bovine MMP-9 fragment, Figure S2: Amino acid sequence of the recombinant Bovine MMP-9 protein from Phe107 to Pro449 (NCBI, NM_174744.2). Figure S3: Purification of GW-H1-IFNγ in *E. coli* BL21(DE3) by IMAC, Figure S4: ICP-MS quantification of metal ions (Zn^2+^ and Ni^2+^) in purified recombinant MMP-9 protein samples [46], Table S1: Impact on bacterial culture growth of MMP-9 gene expression in *L. lactis* and *Clearcoli* [47].

## Appendix A

### Appendix A.1. Bacterial Strains and Plasmids

Electroporation of *Clearcoli* was performed using Gene Pulser from Bio-Rad fitted with 2500 V, 200 Ω and 25 μF in a pre-cooled 2 cm electroporation cuvette. Then, the samples were supplemented with 900 μL of LB medium and incubated for two h at 37 °C. After this, 100 μL of the incubated mixture was plated and incubated overnight at 37 °C.

### Appendix A.2. Protein Purification

Briefly, for *L. lactis*, each 500 mL of the bacterial pellet was suspended in 30 mL PBS containing protease inhibitors (EDTA-free Complete cocktail, Roche, Basel, Switzerland) and was subjected to 4 rounds of cell disruption by French Press at 1500 psi. After cell disruption, lysozyme was added to a final concentration of 0.05 mg/mL and lysates were incubated at 37 °C for 2 h and 250 rpm before washes. In the case of *Clearcoli*, cell pellets were resuspended in 20 mmol/L Tris-HCl pH 8 at 60 mL/g dry weight containing protease inhibitors (EDTA-free Complete cocktail, Roche) and were subjected to 3 rounds of cell disruption by French Press at 1200 psi. Cell lysates were centrifuged at 15,000× *g* for 30 min at 4 °C obtaining supernatant 1 (SN1) and pellet. Pellets were washed twice in Milli-Q water and centrifuged at 10,000× *g* for 30 min at 4 °C (generating samples SN2 in the first wash and SN3 in the second). All supernatants and pellets were stored at −80 °C and saved for further quality control analysis. Pellets were suspended in solubilization buffer (40 mmol/L Tris pH 8 with 0.2% N-lauroylsarcosine) at a ratio of 40 mL per g of pellet and were incubated in agitation (roller mixer) for 40 h (*L. lactis*) and 24 h (*Clearcoli*) at RT. The protein solution was centrifuged at 15,000× *g* and at 4 °C for 45 min, and the supernatant (SN4) containing the solubilized MMP-9 was filtered and purified by immobilized metal affinity chromatography (IMAC) using 1 mL-HiTrap chelating columns (GE Healthcare) in an ÄKTA purifier FPLC system (GE Healthcare). Binding and elution buffers both contained 0.2% N-lauroylsarcosine as well as 20 mmol/L Tris pH 8 and 500 mmol/L NaCl. In addition, binding and elution buffers were prepared with 20 mmol/L and 500 mmol/L imidazole or 10 mmol/L and 500 mmol/L imidazole for *L. lactis* and *Clearcoli,* respectively. The MMP-9 peaks were split by holding the elution buffer gradient at each increase in the absorbance signal in the chromatogram. The eluted peaks were dialyzed separately O/N against 20 mmol/L Tris-HCl pH 8 and 5% glycerol at 4 °C with gentle agitation, centrifuged at 15,000× *g* for 15 min at 4 °C to remove possible precipitated protein and quantified. Aliquots were stored at −80 °C.

### Appendix A.3. MMP-9 Activity Determination by DQgelatinTM Degradation Kinetics

Briefly, for all MMP-9 peaks, 1 μg MMP-9 was plated in a transparent flat-bottom black 96-well plate in triplicate, at a final volume of 150 μL in assay buffer (5 mmol/L CaCl_2_, 50 mmol/L Tris pH 7.6, 150 mmol/L NaCl, 0.01% Tween20). Immediately after adding 0.25 μg of DQgelatin™ per well, the plate was bottom-read every two minutes for 2 h in a fluorescence microplate reader (Victor III multilabel counter, PerkinElmer) at 495/515 nm (excitation/emission wavelengths).

### Appendix A.4. Inductively Coupled Plasma-Mass Spectrometry (ICP-MS) Analysis

Zn^2+^ and Ni^2+^ metal ions present in MMP-9 protein samples were analyzed on an ICP-MS Agilent 7500ce instrument (Santa Clara, CA, USA). Briefly, 100 μL of MMP-9 protein samples in 20 mmol/L Tris-HCl pH 8 and 5% glycerol were dispensed into individual polypropylene tubes in technical duplicates. Protein samples were incubated with 100 μL of HNO_3_ at 80 °C for 30 min. The digested solutions were diluted up to a final volume of 2 mL with deionized water. The samples were analyzed by conventional ICP-MS for the detection of the metal elements Zn^2+^ and Ni^2+^. Sample analysis and operation of the ICP-MS were done according to CCiTUB (www.ccit.ub.edu) in-house standard operating procedures.
