# Supplementary material for: Selecting Subpopulations of High-Quality Protein Conformers among Conformational Mixtures of Recombinant Bovine MMP-9 Solubilized from Inclusion Bodies
Source: Int J Mol Sci. 2021 Mar 16;22(6):3020. doi: 10.3390/ijms22063020 (PMC8001920; doi:10.3390/ijms22063020)
Supplement: Supplementary file 1 [file ijms-22-03020-s001.pdf]

Supplementary material by Carratalá et al.

Title: Selecting subpopulations of high-quality protein conformers among conformational mixtures of recombinant bovine MMP-9 solubilized from inclusion bodies.

Authors: Jose Vicente Carratalá[1,2,3], Laia Gifre-Renom[4], Ramon Roca[4], Julieta Sanchez[1,2,5], Antonio Villaverde[1,2,3], Anna Aris[4], Elena Garcia-Fruitós[4], Neus Ferrer-Miralles[1,2,3]

[1]Institute for Biotechnology and Biomedicine, Autonomous University of Barcelona, Bellaterra, Barcelona, Spain

[2]Department of Genetics and Microbiology, Autonomous University of Barcelona, Bellaterra, Barcelona, Spain

[3]Bioengineering, Biomaterials and Nanomedicine Networking Biomedical Research Centre (CIBER-BBN), Bellaterra, Barcelona, Spain

[4]Department of Ruminant Production, Institute of Agrifood Research and Technology (IRTA), Caldes de Montbui, Barcelona, Spain

[5]Permanent address: National University of Cordoba, Faculty of Exact, Physical and Natural Sciences, ICTA and Chemistry Department, CONICET Institute of Biological and Technological Research (IIByT) Cordoba Argentina

*Corresponding author. E-mail address: neus.ferrer@uab.cat (N. Ferrer-Miralles).

Materials and Methods

Bacteria strains and plasmids

Electroporation of *Clearcoli* was performed using Gene Pulser from Bio-rad fitted with 2500V, 200 Ω and 25 µF in a pre-cooled 2 cm electroporation cuvette. Following, samples were supplemented with 900 µL of LB medium and incubated for 2 h at 37 °C. After that, 100 µL of the incubated mixture was plated and incubated overnight at 37 °C.

Protein purification

Briefly, for *L. lactis*, each 500 mL of bacterial pellet was suspended in 30 mL PBS containing protease inhibitors (EDTA-free Complete cocktail, Roche) and was subjected to 4 rounds of cell disruption by French Press at 1,500 psi. After cell disruption, lysozyme was added to a final concentration of 0.05 mg/mL and lysates were incubated at 37 ºC for 2 h and 250 rpm before washes. In the case of *Clearcoli*, cell pellets were resuspended in 20 mmol/L Tris-HCl pH 8 at 60 mL/g dry weight containing protease inhibitors (EDTA-free Complete cocktail, Roche) and were subjected to 3 rounds of cell disruption by French Press at 1,200 psi. Cell lysates were centrifuged at 15,000 x *g* for 30 min at 4 ºC obtaining supernatant 1 (SN1) and pellet. Pellets were washed twice in Milli-Q water and centrifuged at 10,000 x *g* for 30 min at 4 ºC (generating samples SN2 in the first wash and SN3 in the second). All supernatants

and pellets were stored at -80 ºC and saved for further quality control analysis. Pellets were suspended in solubilization buffer (40 mmol/L Tris pH 8 with 0.2 % N-Lauroylsarcosine) at a ratio of 40 ml per g of pellet and were incubated in agitation (roller mixer) for 40 h (*L. lactis*) and 24 h (*Clearcoli*) at RT. The protein solution was centrifuged at 15,000 x *g* and at 4 ºC for 45 min and the supernatant (SN4) containing the solubilized MMP-9 was filtered and purified by Immobilized Metal Affinity Chromatography (IMAC) using 1 mL-HiTrap Chelating columns (GE Healthcare) in an ÄKTA purifier FPLC system (GE Healthcare). Binding and elution buffers both contained 0.2 % N-Lauroylsarcosine as well as 20 mmol/L Tris pH 8 and 500 mmol/L NaCl. In addition, binding and elution buffers were prepared with 20 mmol/L and 500 mmol/L imidazole or 10 mmol/L and 500 mmol/L imidazole for *L. lactis* and *Clearcoli* respectively. The MMP-9 peaks were split by holding the elution buffer gradient at each increase in the absorbance signal in the chromatogram. The eluted peaks were dialyzed separately O/N against 20 mmol/L Tris-HCl pH 8 and 5 % glycerol at 4 ºC with gentle agitation, centrifuged at 15,000 x *g* for 15 min at 4 ºC to remove possible precipitated protein and quantified. Aliquots were stored at -80 ºC.

MMP-9 activity determination by DQgelatin™ degradation kinetics

Briefly, for all MMP-9 peaks 1 µg MMP-9 was plated in a transparent flat-bottom black 96-well plate in triplicate, at a final volume of 150 µl in assay buffer (5 mmol/L $CaCl_2$, 50 mmol/L Tris pH 7.6, 150 mmol/L NaCl, 0.01 % Tween20). Immediately after adding 0.25 µg of DQgelatin™ per well, the plate was bottom-read every two minutes for 2 h

in a fluorescence microplate reader (Victor III multilabel counter, Perkin-Elmer) at 495/515 nm (excitation/emission wavelengths).

Inductively coupled plasma-mass spectrometry (ICP-MS) analysis.

Zn^{2+} and Ni^{2+} metal ions present in MMP-9 protein samples were analyzed on an ICP-MS Agilent 7500ce instrument (Santa Clara, CA, USA). Briefly, 100 µl of MMP-9 protein samples in 20 mmol/L Tris-HCl pH 8 and 5 % glycerol were dispensed into individual polypropylene tubes in technical duplicates. Protein samples were incubated with 100 µl of HNO_3 at 80 °C for 30 min. The digested solutions were diluted up to a final volume of 2 mL with deionized water. The samples were analyzed by conventional ICP-MS for the detection of the metal elements Zn^{2+} and Ni^{2+}. Sample analysis and operation of the ICP-MS were done according to CCiTUB (www.ccit.ub.edu) in-house standard operating procedures.

Atgg**ca**tttcaaacttttgaaggagaacttaaatggcatcatcataatattacttattggattcaaaattattcagaagatttacctcg

tgctgttattgatgatgcttttgcacgtgcttttgcattgtggagtgctgttactccattaacatttactagagtttatggacctgaagc

agatattgttattcaatttggtgttcgtgaacatggagatggttatccatttgatggaaaaaatggtttgcttgctcatgcatttccacc

tggaaaaggtattcaaggagatgctcattttgatgatgaagaattatggtcacttggaaaaggtgttgttattccaacttattttgga

aatgctaaaggtgctgcatgtcattttccatttacatttgaaggacgttcatattctgcatgtacaactgatggtagaagtgatgatat

gttgtggtgttcaacaactgctgattatgatgcagatcgtcaatttggattttgtccttctgaaagactttatactcaagatggaaatg

ctgatggtaaaccatgtgtttttccttttacatttcaaggtcgtacttatagtgcttgtacatcagatggacgttctgatggttatagat

ggtgtgctacaactgcaaattatgatcaagataaattgtatggtttttgtccaacacgtgttgatgctacagttactggaggtaatgc

tgcaggagaactttgtgtttttccatttacatttttaggaaaagaatattcagcttgtacacgtgaaggaagaaatgatggtcatttat

ggtgtgcaacaacttctaattttgataaagataaaaaatggggattttgtccagatcaaggttatagtttgtttttagttgctgcacat

gaatttggacatgctcttggtttggatcatacttctgttccagaagcattgatgtatcctatgtatcgttttacagaagaacatcctctt

catagagatgatgttcaaggtattcaacatttgtatggaccaagaccagaacctaaacatcatcatcatcatcattaa

Figure S1. *L. lactis* codon optimized DNA encoding sequence of the cloned Bovine MMP-9 fragment. In the DNA sequence design the nucleotides CA following *Nco*I restriction site were added to restore the reading frame. Gene sequence was codon optimized for the *L. lactis* expression host as indicated (Geneart).

MAFQTFEGEL·KWHHHNITYW·IQNYSEDLPR·AVIDDAFARA·FALWSAVTPL·

TFTRVYGPEA·DIVIQFGVRE·HGDGYPFDGK·NGLLAHAFPP·GKGIQGDAHF·

DDEELWSLGK·GVVIPTYFGN·AKGAACHFPF·TFEGRSYSAC·TTDGRSDDML·

WCSTTADYDA·DRQFGFCPSE·RLYTQDGNAD·GKPCVFPFTF·QGRTYSACTS·

DGRSDGYRWC·ATTANYDQDK·LYGFCPTRVD·ATVTGGNAAG·ELCVFPFTFL·

GKEYSACTRE·GRNDGHLWCA·TTSNFDKDKK·WGFCPDQGYS·LFLVAAHEFG·

HALGLDHTSV·PEALMYPMYR·FTEEHPLHRD·DVQGIQHLYG·PRPEP**KHHHH·**

HH

Figure S2. Amino acid sequence of the recombinant Bovine MMP-9 protein from Phe107 to Pro449 (NCBI, NM_174744.2). An Ala residue was added to the original sequence to restore the reading frame (marked in bold). Recombinant MMP-9 gene was C-terminally fused to a His-tag for detection and quantification by western blot analysis. A Lys residue was included at the N-terminus of the tag for putative elimination of the tag by exopeptidases (marked in bold). Structural Zn^{2+}-binding sites are displayed in yellow and Zn^{2+}-binding sites in the active site are displayed in green (UniProt P52176). Trp residues are marked in light grey. MW: 39.60 kDa.

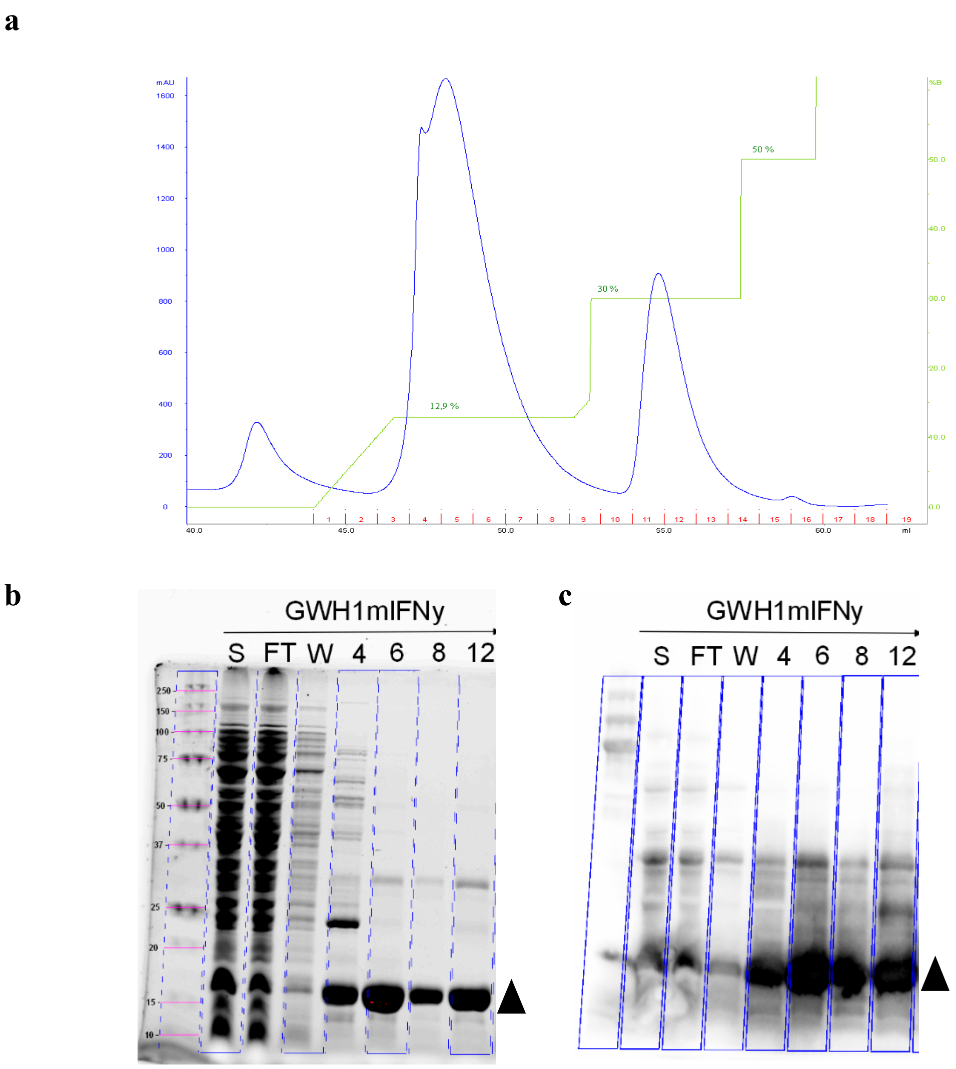

Figure S3. Purification of GW-H1-IFNγ in *E. coli* BL21(DE3) by IMAC. IMAC chromatogram of solubilized GW-H1-IFNγ from IBs. Blue line depicts the absorbance signal (mAU) along the elution process and green line the elution buffer (EB) gradient progress. The corresponding percentage of imidazole (%) is indicated above the green line for each eluted peak (Buffer A: 10 mmol/L imidazole; Buffer B: 500 mmol/L imidazole) (**a**). TGX SDS-PAGE (**b**) and western blot analysis of the purification procedure (**c**). S: proteins solubilized from IBs after N-Lauroylsarcosine incubation; FT: Flow through; W: wash; 4-12: protein fractions. Protein GW-H1-IFNg is a fusion

between an antimicrobial peptide (GW-H1, [46]) and the mouse IFNγ (UniprotKB P01580).

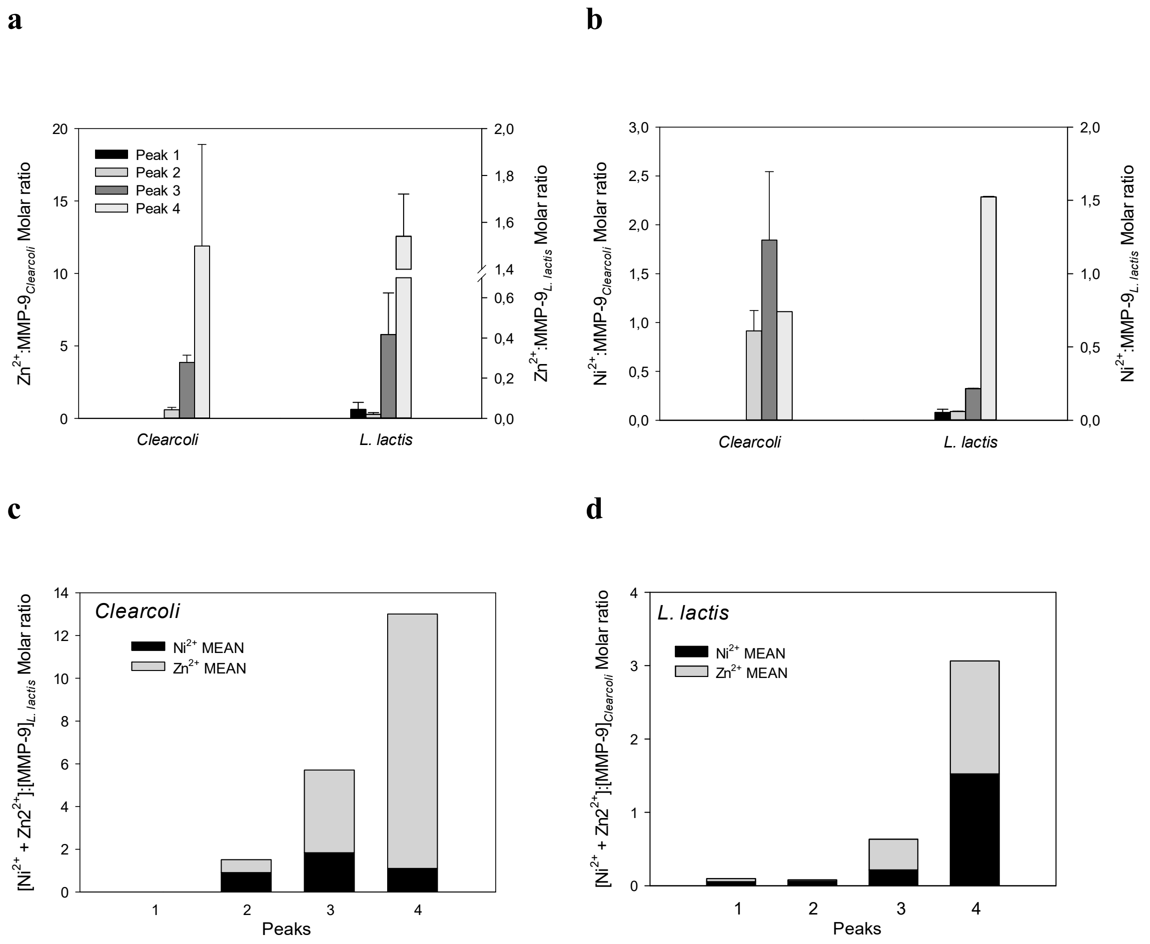

Fig. S4. ICP-MS quantification of metal ions (Zn^{2+} and Ni^{2+}) in purified recombinant MMP-9 protein samples. Molar ratio of Zn^{2+} in protein peaks obtained in *Clearcoli* and *L. lactis* (**a**). Molar ratio of Ni^{2+} in protein peaks obtained in *Clearcoli* and *L. lactis* (**b**). Molar ratio of Ni^{2+} + Zn^{2+} in protein peaks obtained in *Clearcoli* (**c**). Molar ratio of Ni^{2+} + Zn^{2+} in protein peaks obtained in *L. lactis* (**d**). Bars represent mean ± standard error of the mean (**a** and **b**) and mean (**c** and **d**). Protein peak 1 of *Clearcoli* was discarded as the protein purity was < 50 % (see Fig. 2a).

Table S1. Impact on bacterial culture growth of MMP-9 gene expression in *L. lactis* and *Clearcoli*

Host	Code	OD600/550 Pre-inoculum	OD600/550 pre-induction	OD600/550 post-induction	Induction time	Comments
L. lactis	1 (1.5 L)	3.020	0.49 ± 0.024	3.08 ± 0.02	3h 40m	15.9 mg/L (Solubilitzed O/N, 4 ºC)
	2 (2.5 L)	3.015 ± 0.075	0.468 ± 0.015	2.715 ± 0.035	3 h	7.8 mg/L (Solub. 40 h RT)
	3 (2 L)	3.010	0.476 ± 0.0005	3 ± 0.1	3h 10m	9.6 mg/L (Solubilitzed. 40 h RT) **Fractioning error, repeated IMAC for Peak 1**
	4 (2 L)				3 h	8.1 mg/L (Solubilitzed. 40 h RT)
Clearcoli	1 (3 L)	1.975 ± 0.365	0.540 ± 0.007	0.594 ± 0.006	3 h	**Protein samples precipitated after dialysis against 20 mM Tris-HCl pH 8.00 + 5 % glycerol**
	2 (3 L)	1.455 ± 0.145	0.77 ± 0.005	2.786 ± 0.100	18 h	Protein yield was low. **Probable cause: loss of the plasmid**
	3 (3 x 2.4 L)	2.090 ± 0.113	0.568 ± 0.008	0.967 ± 0.078	4 h	0.287 ± 0.011 (mg/L)

As observed in Table S1, expression of MMP-9 gene had a negative effect on *Clearcoli* culture growth. After induction, the growth of the culture stopped (purification 1; Code 1). In addition, longer induction times (purification 2; Code 2) resulted only in tiny amounts of purified recombinant protein despite the higher OD_{550} postinduction measure, suggesting a positive pressure selection of newly appearing plasmid-free cells over the plasmid-bearing initial cell population. It has been widely described that gene expression needs to be tightly regulated for toxic proteins which is not the case of the leaky expression in T7 polymerase system [47].

References

46. Chou, H.T.; Kuo, T.Y.; Chiang, J.C.; Pei, M.J.; Yang, W.T.; Yu, H.C.; Lin, S.B.; Chen, W.J. Design and synthesis of cationic antimicrobial peptides with improved activity and selectivity against *Vibrio* spp. *Int. J. Antimicrob. Agents* **2008**, *32*, 130–138.

47. Giacalone, M.J.; Gentile, A.M.; Lovitt, B.T.; Berkley, N.L.; Gunderson, C.W.; Surber, M.W. Toxic protein expression in *Escherichia coli* using a rhamnose-based tightly regulated and tunable promoter system. *Biotechniques* **2006**, *40*, 355–364.
